# Supplementary material for: DISC1-PML protein interaction for congenital CMV infection-induced cortical neural progenitor deficit: perturbance of host signaling via viral IE1
Source: bioRxiv. 2025 Sep 7:2025.09.03.674025. Preprint. [Version 1] doi: 10.1101/2025.09.03.674025 (PMC12424648; doi:10.1101/2025.09.03.674025)
Supplement: Supplement 1 [file media-1.pdf]

Figure S1

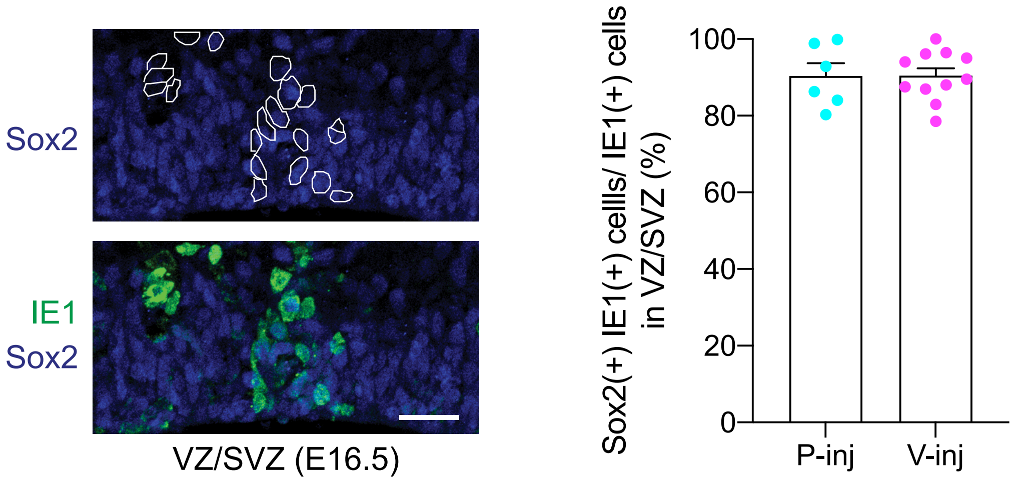
Sox2
IE1
Sox2
VZ/SVZ (E16.5)
Sox2(+) IE1(+) cells/ IE1(+) cells in VZ/SVZ (%)
100
80
60
40
20
0
P-inj
V-inj

Figure S1. IE1 highly localizes in Sox2 positive neural progenitor cells (NPCs) (related to Fig. 1).

Immunostaining for IE1 (green) and Sox2 (blue) in the ventricular zone/ subventricular zone (VZ/SVZ). The white lines indicate the shapes of IE1 immunoreactivity. Scale bar, 50 µm. Graph shows percentage of Sox2 positive cells in IE1 positive cells (mean +/- s.e.m, n=6-11 images). There were no differences in the two models (*P*=0.9805; two-sided Student's *t* test).

Figure S2

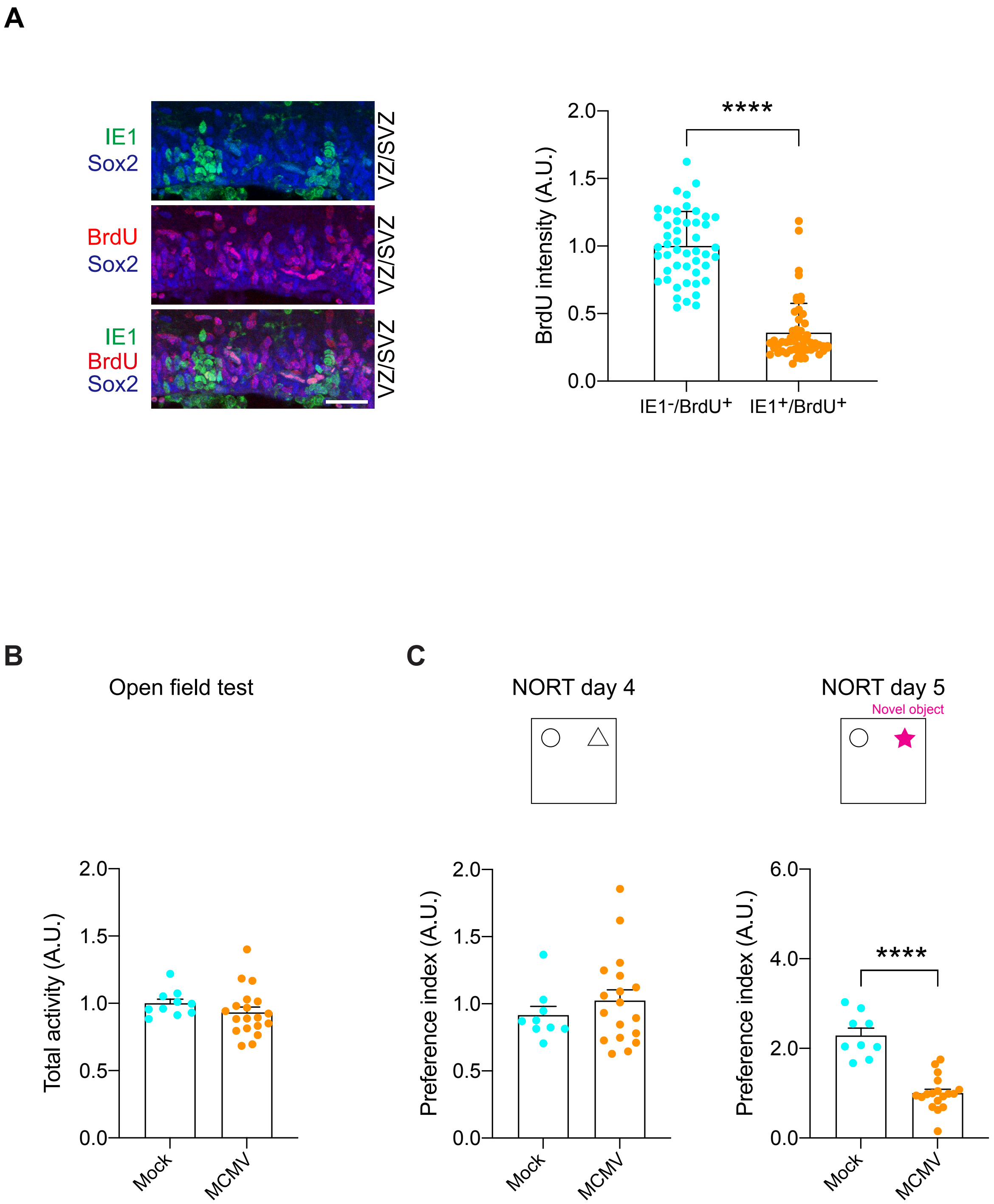
A
IE1
Sox2
BrdU
Sox2
IE1
BrdU
Sox2
VZ/SVZ
VZ/SVZ
VZ/SVZ
BrdU intensity (A.U.)
****
2.0
1.5
1.0
0.5
0.0
IE1$^-$/BrdU$^+$
IE1$^+$/BrdU$^+$
B
Open field test
Total activity (A.U.)
2.0
1.5
1.0
0.5
0.0
Mock
MCMV
C
NORT day 4
Preference index (A.U.)
2.0
1.5
1.0
0.5
0.0
Mock
MCMV
NORT day 5
Novel object
Preference index (A.U.)
****
6.0
4.0
2.0
0.0
Mock
MCMV

Figure S2. Intraventricular injection (V-inj) model shows impairment of neuronal development and a cognitive deficit (related to Fig. 2).

(A) Immunostaining for IE1 (green) and BrdU (red) shows a decrease in BrdU-labeled cells in MCMV-infected areas. IE1-positive nuclei rarely merged with BrdU-positive nuclei in the V-inj model. Scale bar, 50 μm. Graph shows BrdU incorporation in uninfected NPC (Sox2-positive but IE1-negative) and infected NPC (Sox2- and IE1- double positive). The ratio of BrdU- and Sox2- double positive nuclei to total Sox2-positive nuclei was significantly decreased in the infected area compared to the uninfected area. NPC, neural progenitor cell; Blue, Sox2; green, IE1; red, BrdU. Graph shows mean +/- s.e.m. (n=36-55 cells per group, ****P<0.0001; two-sided Student's *t* test).

(B) The total activity of mice infected with Mock and MCMV in the open field test. Total activity was not significantly different between groups. (Mock: n=10, MCMV: n=19; two-sided Student's *t* test).

(C) The results of the NORT in two groups (Mock and MCMV) in the ventricular injection (V-inj) model at 1 month after infection. Graphs indicate the preference index among the groups on day 4 and day 5 of testing. The preference index was calculated as the ratio of exploration time for novel object (or right object on day 4)/exploration time for both objects.

Graphs show mean +/- s.e.m. (Mock: n=9, MCMV: n=18, ***P<0.001, ****P<0.0001; two-sided Student's *t* test).

Figure S3

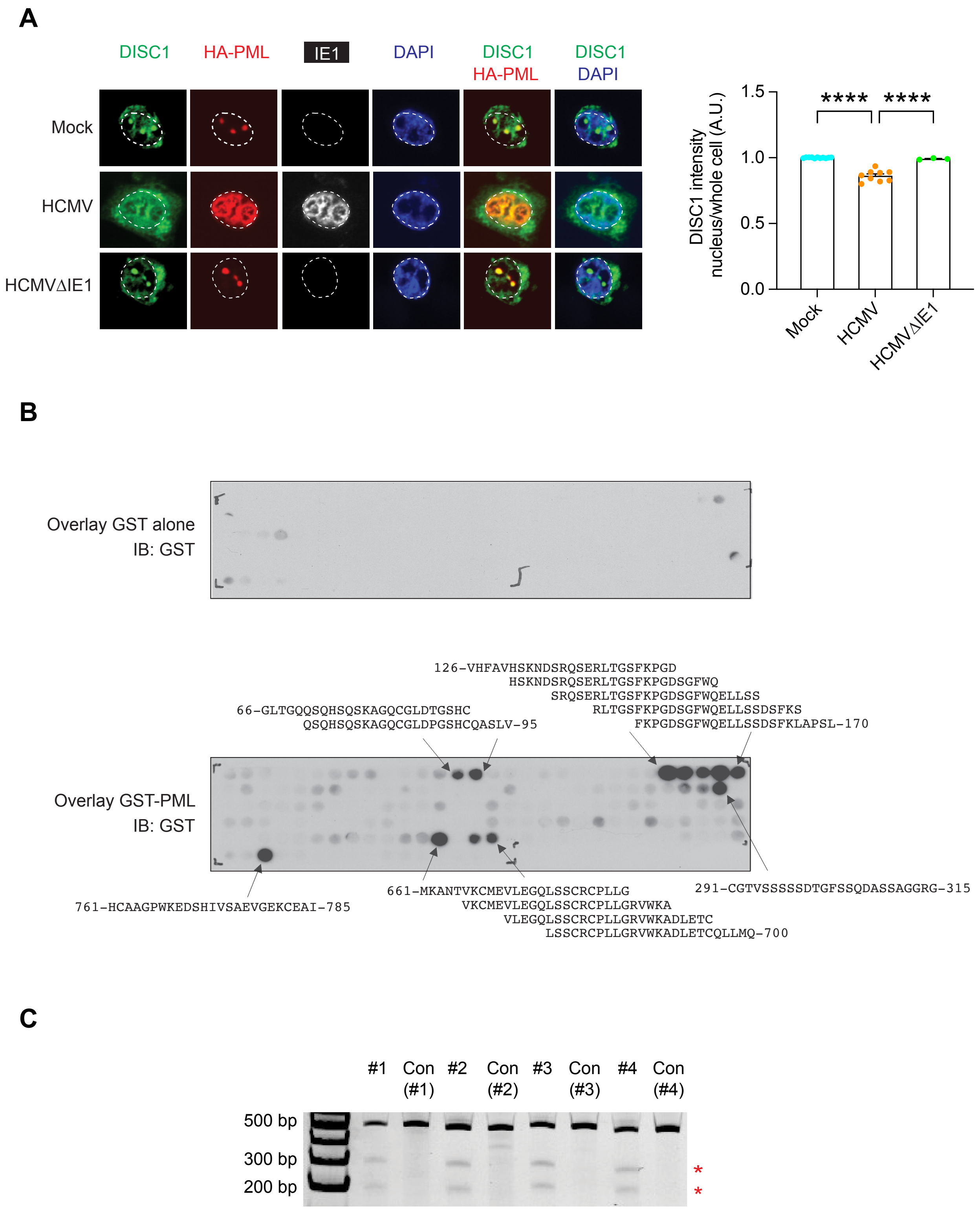
A
DISC1
HA-PML
IE1
DAPI
DISC1 HA-PML
DISC1 DAPI
Mock
HCMV
HCMVΔIE1
DISC1 intensity nucleus/whole cell (A.U.)
1.5
1.0
0.5
0.0
****
****
Mock
HCMV
HCMVΔIE1
B
Overlay GST alone
IB: GST
126-VHFAVHSKNDSRQSERLTGSFKPGD
HSKNDSRQSERLTGSFKPGDSGFWQ
SRQSERLTGSFKPGDSGFWQELLSS
RLTGSFKPGDSGFWQELLSSDSFKS
FKPGDSGFWQELLSSDSFKLAPSL-170
66-GLTGQQSQHSQSKAGQCGLDTGSHC
QSQHSQSKAGQCGLDPGSHCQASLV-95
Overlay GST-PML
IB: GST
761-HCAAGPWKEDSHIVSAEVGEKCEAI-785
661-MKANTVKCMEVLEGQLSSCRCPLLG
VKCMEVLEGQLSSCRCPLLGRVWKA
VLEGQLSSCRCPLLGRVWKADLETC
LSSCRCPLLGRVWKADLETCQLLMQ-700
291-CGTVSSSSSDTGFSSQDASSAGGRG-315
C
#1
Con (#1)
#2
Con (#2)
#3
Con (#3)
#4
Con (#4)
500 bp
300 bp
200 bp
*
*

Figure S3. *In vitro* validations (related to Fig. 3 and Fig. 5)

(A) Co-localization of DISC1-PML in HCMV-infected cells. Images show the nuclei of neuroblastoma cells. Infection with HCMV disturbed PML-DISC1 co-localization in human neuroblastoma. However, infection of HCMV lacking IE1 (HCMV ΔIE1) did not affect the PML-DISC1 co-localization. Green, DISC1; red, HA-PML; white, IE1; blue DAPI. Graph shows mean +/- s.e.m. (Mock: n=11, HCMV: n=9, HCMVΔIE1: n=3, *$P<0.05$, **$P<0.001$; one-way ANOVA).

(B) Pinpointing the PML binding domain on DISC1. DISC1 peptide arrays were probed for PML interaction sites. The dot blot shows interaction between GST-PML and a DISC1 peptide array.

(C) Viral IE1-targeting CRISPR/Cas9 cleaves the IE1 gene. The T7 Endonuclease I assay, which can detect genome cleavage caused by CRISPR/Cas9, shows that the IE1 genes in the MCMV genome are cleaved in MCMV-infected HT22 cells when co-transfected with the IE1-targeting CRISPR construct (IE1-CRISPR). Con, control. Red asterisks indicate cleavage products.

Table S1. Single guide RNA target sequences.

Target	Sequence	PAM sequence
IE1-CRISPR#1	5'- CGGCACGCTCATCTAGTGCG -3'	TGG
IE1-CRISPR#2	5'- AGATTAGTGGGCATGAAGTG -3'	TGG
IE1-CRISPR#3	5'- CACTAGATGAGCGTGCCGCA -3'	TGG
IE1-CRISPR#4	5'- GATGCGCTCGAAGATATCAT -3'	TGG

Table S2. T7 endonuclease I assay primer sequences.

Target	Primer
IE1-CRISPR#1	Fw: 5'- GATATCTTCGAGCGCATCGA -3’<br>Re: 5’- ACACACCCTGTGATATTGG -3’
IE1-CRISPR#2	Fw: 5'- CTGTTGTCCTGTAAGATTGC -3’<br>Re: 5’- CTTCCACCACTACCACATG -3’
IE1-CRISPR#3	Fw: 5'- TCGAAAGACAACGCAAGATG -3’<br>Re: 5’- GGTCTCTAGATGGTCTTTCATG -3’
IE1-CRISPR#4	Fw: 5'- CTCAGTTATTCACATCATGAC -3’<br>Re: 5’- TGTAACAGGGTGGATCATG -3’
